# Migratory movements of Atlantic puffins *Fratercula arctica naumanni* from high Arctic Greenland

**DOI:** 10.1371/journal.pone.0252055

**Published:** 2021-05-28

**Authors:** Kurt K. Burnham, Jennifer L. Burnham, Jeff A. Johnson, Abby Huffman

**Affiliations:** 1 High Arctic Institute, Orion, Illinois, United States of America; 2 Department of Geography, Augustana College, Rock Island, Illinois, United States of America; 3 Department of Biological Sciences, University of North Texas, Denton, Texas, United States of America; MARE – Marine and Environmental Sciences Centre, PORTUGAL

## Abstract

Although the Atlantic puffin *Fratercula arctica* is well studied throughout its temperate and low Arctic breeding range, few have studied the species in its far northern distribution. This study is the first to present data on the migratory movements of the “large-billed” subspecies, *F*. *a*. *naumanni*, that breeds in the high Arctic and which has significantly larger body size than those farther south. During 2013–2015, migration tracks were collected from nine adult puffins (6 males and 3 females) tagged with geolocators in northwest Greenland. Overall, female puffins traveled farther than males on their annual migration, with one female puffin traveling over 13,600 km, which was nearly a third farther than any tagged male in our study. Differential migration was observed in migratory phenology and route, with males using a form of chain migration with acute synchrony between individuals while females appeared to largely use leap-frog migration and showed little synchrony between individuals. Extreme sexual segregation in wintering areas was evidenced by two females that migrated to the southern limit of the species’ range while the six males remained at the northern limit, and wintered along the sea ice edge during portions of the non-breeding season. Male puffins thus wintered in regions with sea surface temperatures up to 10° C cooler than female puffins, and in areas with generally colder sea surface temperatures when compared to previously known wintering areas of temperate and low Arctic puffin breeding populations. The degree to which body size enables male *F*. *a*. *naumanni* to remain in colder waters likely reflects differing life history constraints between sexes and populations (i.e., subspecies). Further study is warranted to investigate how recent changes in climate have further exacerbated the observed differences between sexes in high Arctic puffins and possibly other marine avian species.

## Introduction

Arctic marine and terrestrial environmental conditions such as sea surface temperature (SST), sea ice coverage, and extreme weather are changing with the most rapid and intense shifts occurring in the high Arctic [1]. These environmental conditions are known to influence animal survival and reproduction [e.g., 2–7], and act as migration drivers (for review of drivers of animal migration see [8]). The impact these changing environmental conditions have on animal migratory movements in the low and sub-Arctic region of the North Atlantic are increasingly well studied [e.g., 9–12], however, they are less well studied in the more remote high Arctic where species exist at the most northern edge of their breeding ranges [13–16], where climatic conditions are generally more severe, and where environmental conditions are changing most rapidly [1].

Atlantic puffins *Fratercula arctica* (hereafter puffin) are a charismatic pelagic seabird species of the North Atlantic Ocean. Puffins breed from temperate regions (as far south as Maine, USA (43° N) and Brittany, France (50° N)) to the high Arctic (northwest Greenland (~77.5° N) and Svalbard, Norway (~80° N)), with small populations in northwest Russia and Novaya Zemlya (Russia) (~77.0° N) [17–19]. The species is currently listed as vulnerable, with decreasing colony size and low breeding success reported for many European colonies (which constitutes 90% of all puffins) and the European population is predicted to decline by an estimated 50–79% between 2000 and 2065 [20,21]. Throughout their range the species demonstrates a clinal increase in body size from south to north, with individuals breeding in the far north significantly larger than those nesting farther south [15,18,22]. Additionally, despite an overlap in body sizes between sexes, the species is sexually dimorphic with males on average being heavier (mass) and larger (wing length, bill length, bill depth, etc.) than females [15,18].

**Fig 1:**
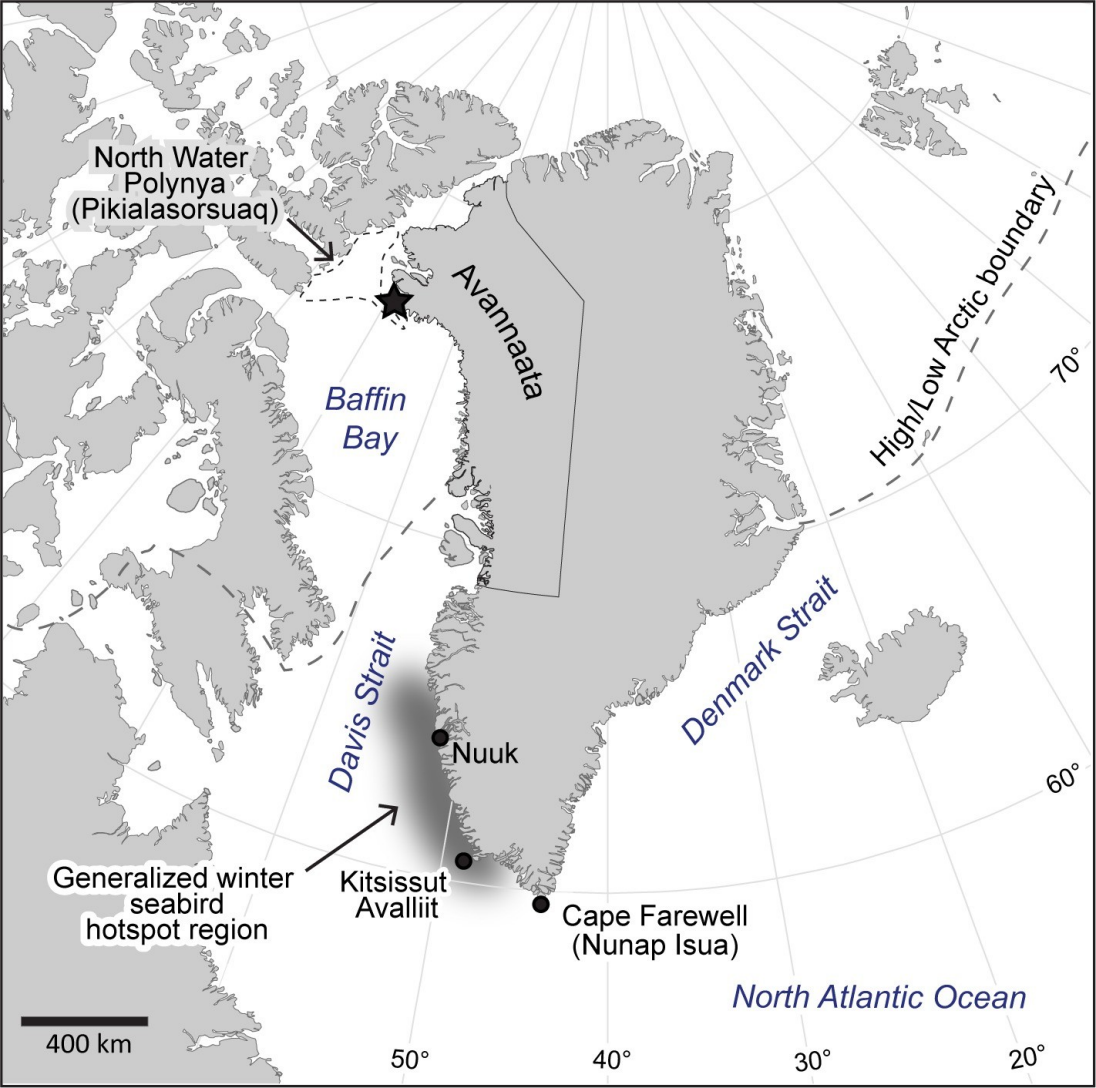
Study area map and geographic place names. Map indicating location of study colony (black star) and place names used throughout the paper. Country boundaries from [28].

Greenland has an estimated 5000 pairs of puffins. The population is considered “near threatened” and appears to be in decline [23]. The breeding range is predominantly on the west coast and extends from Kitsissut Avalliit (60° N) in the southwest to the high Arctic in the northwest (77.5° N) (Fig 1) [24–26]. Aside from Salomonsen’s [26] general description of the puffin in 1950 and a recent study describing morphology differences in Burnham et al. [15], limited information exists on the biology of puffins in Greenland, with sporadic and anecdotal information provided in the literature on colony size and location. Only a single colony-specific study has been published that we are aware of [15] and apart from a single band recovery from a puffin chick originating in Nuuk (64.10° N, Fig 1) in July 1954, and recovered in Conception Bay, Newfoundland in December, 1954 ([27]; P. Lyngs pers. comm. 2019), no information exists about winter movements of puffins breeding in Greenland.

The high Arctic of northwest Greenland is home to the most northern population of puffins in the western North Atlantic with an estimated 150–250 pairs distributed among ~8 colonies ([15,29]; K. Burnham unpubl. data). The population is considered to be the high Arctic “large-billed” *F*. *a*. *naumanni* subspecies, which is also in Svalbard (excluding Bjørnøya) and Novaya Zemlya, and is characterized by its extremely large body size compared to the other puffin subspecies [15,18,30,31]. Puffins belonging to the *naumanni* subspecies in northwest Greenland are among the largest puffins documented, and are significantly larger (mass, bill, wing) than puffins in populations farther south in temperate and low Arctic areas [15]. However, extreme variation in body size exists both within and between sexes in this population when compared with more southern populations, and the largest males are over 50% heavier than the smallest females [15]. The reason(s) behind these differences are unknown, but environmental conditions may play an important role.

Increasingly, research on puffins has focused on the migratory strategies and drivers of adult movement during the non-breeding season and the large degree of variation that exists [32–37]. These strategies can include dispersive [8] or directional migration [9], which can be driven by factors such as food availability, environmental conditions, competition, and sex, and frequently a combination of drivers play a role (see [38] for review). For example, seabirds breeding in the most northern regions of the high Arctic are forced to migrate as a result of multiple months of almost total darkness and a complete lack of prey due to sea ice coverage, with open water in some cases well over a thousand kilometers away from their breeding territories. Drivers and pressures vary frequently between seabird colonies, resulting in differences in migratory phenology, route, and wintering area between populations [e.g., 39–43]. Similar differences in migratory patterns also occur within colonies, and can be a result of sexual segregation, frequently due to differences in body size [e.g., 44–46]. For example, the body size hypothesis suggests that larger individuals are better able to survive in colder temperatures and more harsh environments, as may arise in sexual dimorphic species [47–49].

Puffin migration studies to date have focused only on breeding colonies from temperate and low Arctic Europe and eastern North American regions (for review see [50]). No information has been published on breeding colonies in the high Arctic that experience more harsh environmental conditions. Here we provide results from a three-year geolocator study of Atlantic puffins that breed in high Arctic northwest Greenland. The goals of the study were to: 1) identify migratory routes and phenology, and determine non-breeding spatial distribution patterns, 2) compare migratory behavior and spatial interactions with puffins from other colonies across the North Atlantic, and 3) identify possible migratory drivers of non-breeding season spatial distributions.

## Materials and methods

Ethics Statement: All applicable ethical guidelines for use of birds in research were followed, including those presented in the Ornithological Council’s *Guidelines to the use of wild birds in research* [51]. All research in Greenland was conducted after ethical approval and issuance of permits by the Government of Greenland, Department of Fisheries, Hunting and Agriculture (High Arctic Institute permit numbers: Sags nr. 2013–083369, Dok. nr. 1204884, Sags nr. 2014–099682, Dok nr. 1594176, Sags nr. 2015–115204, Dok. nr. 1975643). Bird bands were provided by Zoological Museum, Natural History Museum of Denmark, University of Copenhagen.

Fieldwork on puffins was carried out during their breeding season from 2013–2015 at Dalrymple Rock (Igánaq) (76.47° N, 70.22° W), a small island in the high Arctic municipality of Avannaata in northwest Greenland (Fig 1). Weather during the breeding season can be severe, with snow possible in every month and wind speeds in excess of 45 m/s [13]. Air temperature during the breeding season averages 3.5° C, with June being the coolest (2.5° C) and July the warmest (5.4° C) (calculated from [52]). Summer ice-free SST in northern Baffin Bay ranges from 1–4° C [53], with a long-term warming trend observed [54]. Sea ice breakup in the study area does not begin until May and refreezes by mid-October [55]. Sea ice coverage expands southward through the Davis Strait from October to March [55], limiting prey availability and thus forcing puffins to migrate south. There was no indication that puffins from this study occupied the open water of the nearby North Water Polynya (Pikialasorsuaq) (Fig 1) during fall or winter.

The colony has an estimated 15–35 pairs, with puffins arriving at the start of June and departing by mid-to-late September ([15]; K. Burnham unpubl. data). Due to near surface permafrost, puffins on Dalrymple Rock are unable to excavate burrows and nest only in crevices or under boulders [15,26]. Information on breeding chronology is limited to a single chick that fledged on 13 September in 2017 as captured on a camera trap (K. Burnham unpubl. data).

Adult puffins were captured at the colony using a combination of dip nets and noose carpets. After capture, puffins were banded (Danish government leg band and Darvic color band), weighed [15], sampled (feathers and blood), aged (adults possess two or more bill grooves) [18], tagged with an archival light tag (hereafter referred to as geolocators), and shortly after release were observed entering and exiting nests. Puffins were sexed using a standard molecular method as described elsewhere [56] using primers 2550F and 2718R [57] and DNA extracted from either blood or breast feathers with the DNeasy Blood and Tissue Kit (Qiagen Inc.). No information was collected on breeding success although individuals were regularly observed entering and exiting their apparent nests. No adults were observed returning to the colony with fish in their bills during our fieldwork at the breeding colony, suggesting that our visits took place during the incubation period.

From 11 to 24 July in 2013 and 2014 a total of 25 Mk4093 geolocators (1.5 g, Biotrack, United Kingdom) were deployed on adult puffins via attachment to a colored Darvic leg band using a cable tie and amalgamating tape [58]. The weight of the geolocator, band, cable tie, and tape combined (2.4 g) were less than 0.6% of total puffin body mass at time of initial geolocator deployment (mean ± standard deviation (sd) = 559.0 ± 64, range = 426–716, *n* = 23). The geolocators recorded ambient light data and wet/dry data, but not temperature data, and had an expected battery life of over two years. For puffins re-captured after a single year, data were downloaded from the geolocator when possible and the bird was released with the original unit in place to collect an additional year’s movement information or until the battery no longer functioned. In two instances the geolocator was removed and replaced with a new unit. The handling process to deploy birds with geolocators took 8 to 12 minutes (min) and up to 20 min for birds that required data download while the geolocator remained on the bird. Visits to the colony were limited in both overall duration and frequency to minimize disturbance.

Light data from geolocator units was processed using the BASTrak software package (British Antarctic Survey, Cambridge, UK) using a light threshold of 10. Determination of sun angle was made using control data from units left on site for one year. A sun elevation angle of -3.0 was used for all but one geolocator (-2.5; a male), which resulted in the smallest location bias. Wet/dry sensor data were utilized to verify plausibility of land-based migratory pathways and to validate appropriate sun angle selection, and in a single instance a sun angle of -2.5 was used to provide a more realistic path aligned with wet/dry sensor data. Data were filtered using a minimum dark period of 4 hr. Geolocators record ambient light, from which a sunset and sunrise time are determined, and thus data were excluded during periods of 24 hr daylight in June, July, and August (see [59] for more information on geolocators). Resulting locations were smoothed in R (R Core Team, Vienna, Austria) using a three-position moving average based on spherical trigonometry [60] to reduce the impact of outliers on our distance calculations since geolocators have substantial errors that have been previously estimated at up to 185 km [59]. Positions recorded on dates 15 days on either side of the spring and fall equinox were removed because locations are likely unreliable during that time period due to weak latitudinal trends in day length [36,59]. Filtering was applied to remove points with unrealistic movements (i.e., movement > 500 km/day) and points were visually inspected for accuracy and plausibility [35]. Only inland points greater than 100 km from the sea were removed to avoid offshore distribution bias [61,62]. ArcGIS 10.7.1 (ESRI, Redlands, CA, USA) was used for display, analysis, and distance measurements.

Tri-monthly median locations (three locations per month) for each bird were calculated using ArcGIS for 10-day periods between October and May with months divided into three equal parts (e.g., 1–10 Dec, 11–20 Dec, 21–31 Dec); however, not all 10-day periods had equal number of days due to removal of anomalous data as described above. Daily movements described in the results are thus not always visible in presented figures as a result of tri-monthly median location use and this is noted when applicable. Tri-monthly medians were used instead of kernel density estimation as a result of the limited number of geolocators retrieved, the long distances traveled relatively quickly by some individuals (which would have likely been obscured if kernel density had been used), and to better highlight the differences between sexes, years, and individual puffins.

Total migration distance was calculated by summing the great-circle distances (GCD) from the colony to the first tri-monthly median location, from the first to second location (and so on), and the last median location back to the colony [50]. Mean latitude and longitude of the eight most distant validated locations from the colony (not always in the same month) were used to calculate the farthest GCD from the colony, and all validated points within a month were used to calculate monthly mean GCD from colony. When calculating total migration distance, farthest GCD from colony, and mean monthly GCD from colony, adjustments were made if an individual appeared to cross the Greenland Ice Sheet. The shortest possible at-sea route around the southern tip of Greenland was used since puffins are not thought to travel great distances over land [50]. The start of return migration was defined as when an individual began moving northward in the general direction of the breeding colony.

For spatial comparisons between colonies, data from Fayet et al. [50] (Figure S1, F–J) were georeferenced in ArcGIS and the mapped maximum monthly occupancy kernels for each colony were traced as continuous polygons. Maximum monthly occupancy kernels for colonies overlapped in some instances and thus individual polygons may represent more than one colony. Furthermore, some colonies are represented by more than one polygon during any given month. These data are thus modified and are not identical to the original image. For examination of potential environmental migratory drivers, maximum sea ice extent [55] and SST [53] were overlaid with puffin spatial locations in ArcGIS and observed patterns were noted and described.

Data were analyzed using Minitab 19 (Minitab, Inc., State College, PA, USA). Only descriptive statistics are provided due to the small sample size of adult female puffins.

## Results

Geolocators were recovered from 9 of the 23 tagged individuals (39%) and an additional seven tagged adults were observed but not re-captured for a total of 70% return rate. Recovered geolocators yielded single years of data for six individuals (4 males, 2 females) and two years of data for three (2 males, 1 female) (Table 1). Units on females 8408 and 7314 failed part way through deployment and provided only partial years of data, however, the failed geolocator on female 8408 was replaced with a new unit in 2014, thus resulting in a partial year of data in year one and a complete year of data in year two (Table 1). Mean mass of males and females at initial capture, and for which complete migrations were recorded, was 598 ± 36 g (*n* = 6) and 441 ± 21 g (*n* = 2), respectively, and males were on average 157 g heavier than females (Table 1). Notable differences in migratory phenology, route, and distance were observed between male and female puffins and among females, but not among males.

**Table 1:** Total migration distance and farthest distance from breeding colony for nine Atlantic puffins breeding at a colony in northwest Greenland.

Puffin	Sex	Year	Total migration distance (km)^a^	Farthest distance from colony (km)^b^	Farthest location from colony^c^	Period farthest from colony	Mass (g)^f^
8406	F	2013/14	13,670	5199	34.1° N, 27.8° W	5–21 Apr	426
7360	M	2013/14	8341	2549	56.8° N, 39.0° W	9 Apr–2 May	626
		2014/15	7103	2401	58.4° N, 37.8° W	16–25 Feb	
7363	M	2013/14	8504	2443	60.6° N, 25.6° W	5–17 Apr	546
		2014/15	7867	2780	56.9° N, 27.0° W	5 Apr–3 May	
7314	F	2014/15^d^	5784	2229	59.6° N, 39.9° W	14–25 Nov	542
8408	F	2013/14^e^	7670	4473	41.7° N, 23.8° W	9–26 Jan	456
		2014/15	10,261	4463	38.4° N, 50.2° W	8 Apr–13 May	
8413	M	2014/15	8562	2644	55.6° N, 41.0° W	14–26 Apr	602
8420	M	2014/15	7490	2914	53.5° N, 38.6° W	11–22 Apr	627
8422	M	2014/15	7030	2372	58.3° N, 40.0° W	7–17 Apr	627
8427	M	2014/15	8896	2821	54.7° N, 36.2° W	5–18 Apr	562

^a^ sum of distances to and from colony and tri-monthly median locations from recorded geolocator data entry points

^b^ mean great-circle distance of the eight farthest locations from the breeding colony

^c^ mean of eight farthest locations from breeding colony

^d^ incomplete migration, geolocator data only available through mid-November

^e^ incomplete migration, geolocator data only available through January in 2013/14

^f^ mass when initially tagged with geolocator

### Outward migration

**Fig 2:**
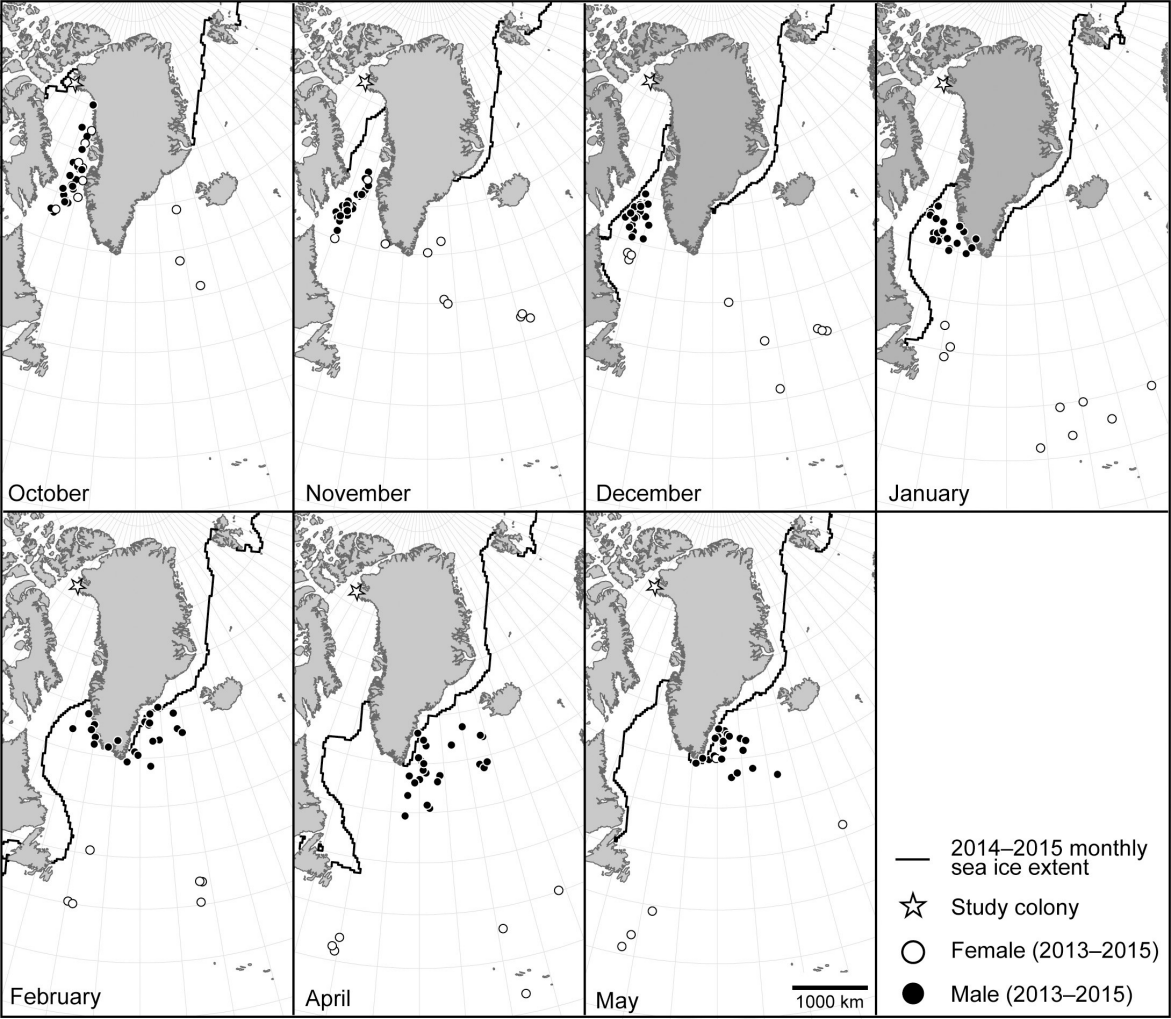
Tri-monthly median locations of nine adult Atlantic puffins from northwest Greenland. Monthly maps indicate segregation of puffins by sex during the non-breeding season with each individual bird represented by a maximum of three points per month. Locations on the Greenland Ice Sheet are a result of individuals rapidly transiting around the southern tip of Greenland from the southwest to southeast, thus resulting in a median location on the Greenland Ice Sheet. Data source for country boundaries is [28] and monthly sea ice extent data is from [55].

**Fig 3:**
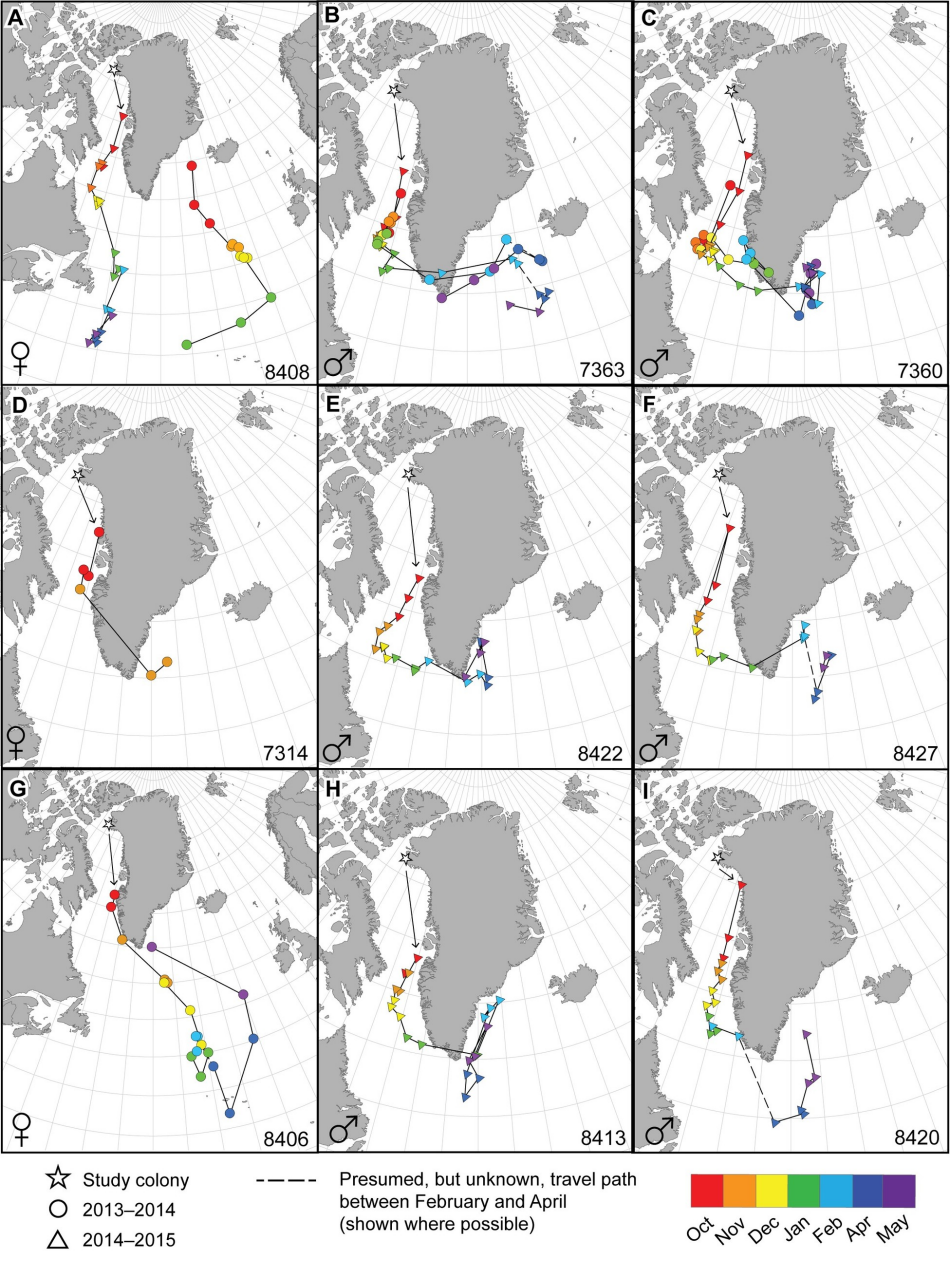
Migratory paths of nine individual Atlantic puffins tagged in northwest Greenland. Each color-coded month has two or three median locations (e.g., 1–10 Dec, 11–20 Dec, 21–31 Dec). Panels A–C include locations for individuals with two years of data and panels D–I have individuals with a single year of data. Locations and migratory routes on the Greenland Ice Sheet are a result of individuals rapidly transiting around the southern tip of Greenland from west to east resulting in the median location or path crossing the Greenland Ice Sheet. No data for September or March were included due to equinox periods making location identification problematic. Country boundaries from [28].

Male puffins had similar migratory phenology and routes during outward migration. All six males (and two males in multiple years) were in southern Baffin Bay in mid-October and had moved south to the Davis Strait by month’s end (Figs 2 and 3). Through November and December, the male puffins congregated along the northern edge of the Labrador Shelf and the encroaching sea ice edge (Figs 2 and 3). Between late-January and the end of February the majority of males moved rapidly from the Davis Strait to southeast Greenland or the Denmark Strait (Figs 2 and 3). The remaining males likely followed a similar pattern, but their proximity to the spring equinox produced unrealistic movement patterns and their positions during that time period were excluded from the analysis. By early April all males had moved into the North Atlantic, south of Greenland or Iceland (Figs 2 and 3). All but one male was at its farthest point from the colony in April and the most southern migration point for males ranged from 53.5° N to 60.6° N (Fig 3; Table 1). During this time period males appeared to be more dispersed from one another than at any other period of the study.

The two male puffins with two years of data (7363 and 7360) showed low between-year variation in migratory phenology and route (Fig 3B and 3C). The average distance variation between tri-monthly median locations for males 7363 and 7360 was 262 km (median = 242 km, *n* = 19, sd ± 151 km, range = 56–597 km) and 308 km (median = 249, *n* = 20, sd ± 249 km, range = 19–1005 km), respectively. The larger average, standard deviation, and range for male 7360 was the result of a small reverse migration north prior to transiting Cape Farewell (Nunap Isua; most southern point of Greenland) during the spring equinox (March) in 2013/14 versus transiting Cape Farewell in late January to early February in 2014/15 (Fig 3C).

Migration patterns for the three female puffins varied widely. One female (8406) departed the breeding area and traveled rapidly south along the west coast of Greenland. This female crossed Cape Farewell and traveled southeast in early-to-mid November, until it was approximately 5200 km from the colony at ~34.1° N, in the mid-Atlantic Ocean south of the Azores in April (Fig 3G; Table 1). A second female (7314) was in southern Baffin Bay in early October and by mid-October had transited Cape Farewell to southeast Greenland, ultimately reaching northwest Iceland at the end of January (destination obscured on map through use of tri-monthly median points) where at that point the geolocator failed to record additional data (Fig 3D).

The third female (8408), which had two years of data, showed distinct variation in migratory phenology and route between years and between the other two females. In year one (2013/14), the female was first recorded in the Denmark Strait at the start of October and then moved southeast to an area northeast of the Azores, where it reached as far as ~20° W, the farthest east of any puffin in this study (Fig 3A). From there it began rapidly moving west, after which the geolocator failed and no additional data were recorded (Fig 3A). Throughout year two (2014/15) the same female remained in the western North Atlantic for the entire non-breeding season. It was in southern Baffin Bay in early October and moved south to ~700 km off the coast of Newfoundland, Canada, where it spent the month of April and first half of May (Fig 3A; Table 1). In both years the farthest distance from the colony was approximately 4500 km and the most southern latitude reached was similar (41.7° N vs. 38.4° N), however, the distance and latitude was reached in January 2014 (no data after this point during 2013/14) and in 2014/15 it was not reached until mid-April (Fig 3A: Table 1). Over 15° of longitude separated these most distant points, but the female in 2013/14 was moving rapidly west in the general direction of the April 2014/15 location when the geolocator failed in January (Fig 3A).

### Return migration

Male and female puffins generally reached the farthest average distance from their breeding colony in mid-April (Figs 2 and 3; Tables 1 and 2). All six males (two in multiple years) initiated return migration between late April and early May, congregating off the coast of southeast Greenland in an apparent staging area, where they remained until mid-May. Four individuals moved southwest from this congregation point between 21 and 31 May and appeared to start transit around Cape Farewell, although this is not apparent in Fig 3 due to the use of tri-monthly medians. It is likely that all males followed a similar path, but the time period coincided with the onset of continuous sunlight which influenced data reliability for several birds. The two males with two complete years of data (7360 and 7363) were in the same general location off the coast of southeast Greenland in both May of 2014 and 2015 (Fig 3B and 3C).

**Table 2:** Mean monthly distance of male and female puffins from the breeding colony in northwest Greenland.

	Oct	Nov	Dec	Jan	Feb	Apr	May
Male	1207±169, 908–1459, *n* = 8	1444±178, 1160–1654, *n* = 8	1595±125, 1440–1777, *n* = 8	1799±148, 1520–1938, *n* = 8	2521±570, 1617–3005, *n* = 8	2890±367, 2509–3458, *n* = 8	2798±245, 2558–3269, *n* = 8
Female	1701±1006, 998–3194, *n* = 4	2724±887, 1635–3795, *n* = 4	3189±987, 2085–3987, *n* = 3	3885±765, 1938–4441, *n* = 3	3672±375, 3406–3937, *n* = 2	4581±109, 4509–4653, *n* = 2	3614±376, 3348–3880, *n* = 2
Difference male vs female	494	1280	1594	1952	1698	1691	1249

Values for male and female presented as mean ± sd, range, sample size. Distances given in km.

The two females with available return migration data (8406 and 8408 [2014/15]) were over 2000 km farther from the colony than males at the start of return migration (Figs 2 and 3A and 3G). In late April female 8406 was over 2500 km southeast of the males and began rapidly moving north. By mid-May 8406 had joined the males off the coast of southeast Greenland (Figs 2 and 3). Female 8408 did not start moving north until the end of May when it was southeast of Newfoundland (Fig 3G), and as with males, the northern movement was somewhat obscured in Fig 3 by using tri-monthly medians.

### Total migration distance, distance from colony, and latitude

Female and male puffins traveled an average total migration distance of 11,966 km (*n* = 2, sd ± 2411 km, only years with complete tracks) and 7974 km (*n* = 8, sd ± 708 km, includes repeated measures from two males), respectively (Table 1). Females traveled approximately one-third farther than males and female 8406 traveled the farthest at 13,670 km, or >3400 km more than the other female. Less variation in total migration distance was observed among males with an approximate 1900 km difference between the shortest and longest of the eight complete migrations.

Farthest distance traveled from the colony for puffins with complete migration tracks showed females traveled almost twice as far as males. Females achieved a mean distance of 4831 km (sd ± 520 km, *n* = 2, range = 4463–5199 km) at their farthest point from the colony while males traveled a mean distance of 2616 km (sd ± 207 km, *n* = 8, range = 2401–2914 km). Latitudinally, the average farthest position reached by the two females with complete migration tracks was 36.3° N (sd ± 3.0°, range = 34.1–38.4° N) while males traveled only to an average 56.8° N (sd ± 2.3°, *n* = 8, range = 53.5–60.6° N), over 20° farther north than females (Table 1).

Monthly mean distance from the colony varied widely between the sexes (Table 2). The average difference between males and females was smallest in October (494 km) and largest in January (1952 km).

Although the small sample sizes prevented us from making statistical comparisons between sexes, the observed patterns do suggest that differences between sexes exist. Additional study is warranted with larger sample size to verify that consistent patterns exist.

### Inter-colony spatial comparisons

**Fig 4:**
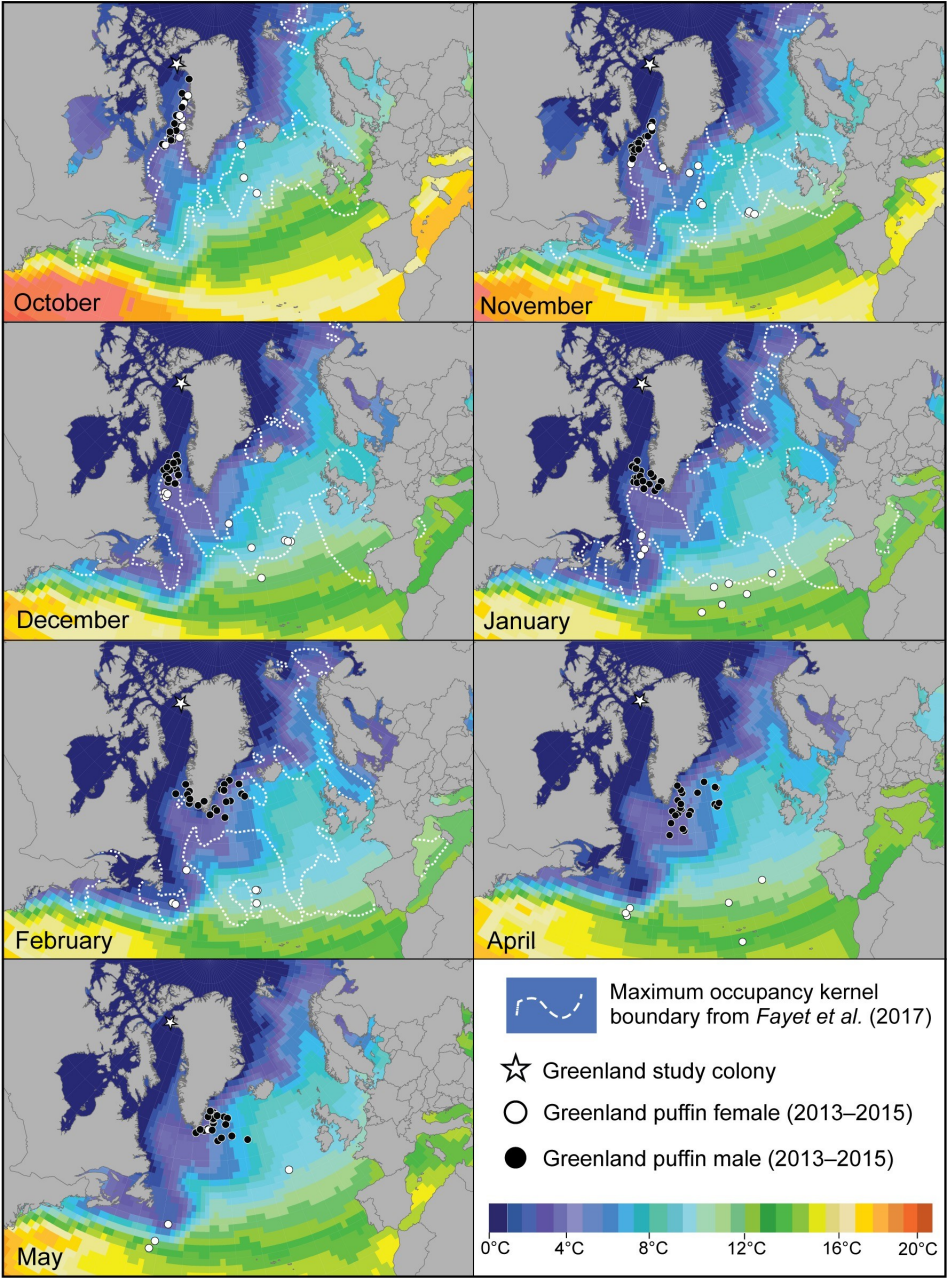
Atlantic puffin monthly winter range and sea surface temperature. Tri-monthly median locations of puffins from northwest Greenland and monthly puffin winter use areas from Fayet et al. [50] overlaid with 2013/14 average monthly sea surface temperatures [53]. No use area data for April/May was available from Fayet et al. [50] because puffins began returning to breeding colonies at that time, and no data for September or March for either study were available due to equinox periods. Country boundaries from [28].

Puffins from northwest Greenland generally wintered in different areas than those from colonies in temperate and low Arctic North America and Europe (Fig 4). Male puffins predominantly remained north of other known puffin wintering areas while females overlapped with other populations from October to December, and then moved south of them. Variation was also apparent between colony winter use areas and SST. Male puffins wintered in mostly colder waters than females and puffins from other populations.

## Discussion

Atlantic puffins breeding in northwest Greenland (76.5° N) traveled between 7000 and at least 13,700 km during their annual migration, and the southern extent of their migration ranged from 34–61° N in the North Atlantic. The two females traveled to significantly more southern latitudes and farther than all six males, which is reflected by differences in migratory phenology, route, and wintering area between the sexes. This is the first migratory study of puffins breeding in northwest Greenland, and the high Arctic in general. Below we discuss the distinct differences between our study population and previously studied puffin populations in temperate and low Arctic regions and what role body size and environmental conditions may have played in our findings.

### Comparison of migratory strategies between colonies

Puffins from northwest Arctic Greenland have the longest total migration distance recorded to date of any puffin breeding colony. When compared to the two puffin colonies in Ireland and Norway that previously had the longest average migration distances (4736 ± 238 km and 4465 ± 357, respectively [50]), Greenland males (*n* = 6) traveled almost 3000 km farther and females (*n* = 2) over 7000 km farther. However, data from puffins in Ireland and Norway were collected from 2010 to 2013 [50] and 2012 to 2015 [50], respectively, and variation in environmental conditions across years could account for some of the observed difference. No migration data from other high Arctic puffin breeding colonies have been published to date, and therefore no comparisons can be made with other high Arctic *F*.*a*. *naumanni* populations.

While we demonstrate sex-specific differences in migratory phenology, route, and distance in puffins from northwest Greenland, such differences have been observed infrequently elsewhere throughout their distribution. Fayet et al. [36] found that sex was not correlated with migration route for puffins tagged on Skomer Island, Wales, UK; however, females remained significantly closer to the colony than males in November–January. The mean difference in distance from the colony between sexes was ~200 km in the Fayet et al. [36] study, whereas in our study, females were an average of 494 km farther from the colony than males in October and progressively increased to nearly 2,000 km in January, and remained over 1,000 km into May. The only other studies to investigate sex-specific differences occurred at colonies in southwest Ireland [35] and southeast Scotland [33], and no differences were found.

Both Guilford et al. [34] and Fayet et al. [36] found that male and female puffins showed strong consistency in migratory routes between years. Although males in this study did show high levels of consistency between years, the migratory phenology and route used by the single female with two years of data were different between years. Guilford et al. [34] found puffins from Skomer Island had a dispersive migratory strategy, with no general wintering area. The females from our study could also be described as dispersive (longitudinally), but male puffins possessed a strong degree of similarity in both migratory phenology and overwintering location. Additional female samples are needed to determine if the observed pattern is consistent among female puffins within northwest Greenland.

Female and male puffins from northwest Greenland showed distinct differences in overlap with the winter ranges of North American and European puffin populations. Male puffins from northwest Greenland spent October through December just to the north of puffins from Iceland, occupying an area near southwest Greenland, a known seabird hotspot [63–66] (Figs 1 and 2). With the exception of some minor overlap, Greenland male puffins generally remained north of areas where Icelandic puffins congregated and did not share the same area based on our location data (Fig 4). This general pattern continued from January through February, with male puffins from northwest Greenland only moving into known puffin use areas once they had been vacated by puffins from other geographic populations, or into areas shown to be sparsely or not used by puffins from other populations ([47]; Fig 4). Only in April, when puffin populations studied by Fayet et al. [50] began returning to their breeding colonies, did male puffins from our study colony disperse to the most southern and eastern extent of their wintering area (Fig 4). Throughout the non-breeding season, male puffins from northwest Greenland remained farther north and generally separate from other overwintering puffin populations, only moving into new areas after puffins from other breeding colonies had departed (Fig 4). This migratory pattern is strongly suggestive of a form of chain migration (see [67]), which has not previously been described in puffins and is uncommon in seabirds (but see [68]), and would reduce intraspecific competition for prey.

In contrast, as female puffins from northwest Greenland migrated south, they overlapped in their use areas from October to December with puffins from Iceland, Celtic Sea, and Ireland ([50]; Fig 4). From January onward they demonstrated minimal overlap, and migrated south of puffins included in Fayet et al. [50] to what is generally considered the southern limit of the species’ non-breeding range, south of the Azores and far off the coast of the northeast United States [18,19,21]. This migratory pattern fits well with the description of leapfrog migration, which is when individuals that nest at higher latitudes over-fly populations breeding at lower latitudes to winter farther south, and similar to chain migration would thus reduce intra-specific competition for prey [67]. Although leapfrog migration has been described in other seabird species (see [69,70]), the migratory behavior is generally uncommon. This pattern has not been shown in puffins; however, our sample size for female puffins is small and additional samples from northwest Greenland are needed to confirm this pattern.

### Return migration

Tri-monthly median locations showed that most puffins congregated off the coast of southeast Greenland and started their return migration at the end of May in 2014 and 2015. Camera traps at the study colony documented puffins first arriving on 9 June in 2018 and 2 June in 2019 (K. Burnham unpubl. data; includes puffins tagged with geolocators), indicating a rapid return migration rate. Based on this information it seems likely that puffins traveled the ~2000 km distance to the breeding colony in a week or less, which fits with the generally accepted maximum daily flight distance of 500 km per day for puffins (8 hr of sustained flight at a mean speed of 64 km/h, [36, 71]). This general pattern of a more rapid return migration than outward migration has also been found in many other long-distance migrants, including seabirds [e.g., 72,73]. There was no indication that males arrived earlier at the breeding colony than females despite wintering much closer, although as previously mentioned, our samples size for females was low.

### Use areas of geographic importance

During our study, male puffins spent the majority of December along the sea ice edge, with more limited use from January through May (Fig 2). The importance of this over-wintering habitat for both birds and mammals is well documented [74–76]. Recent advances in tracking technology have increased our knowledge on how these areas are used and have documented additional species previously not known to utilize that habitat [e.g., 77,78]. For example, some gyrfalcons *Falco rusticolus* that breed in northeast Greenland spend their winter months along the sea ice edge in the North Atlantic likely due to the high abundance of overwintering seabirds as prey in the same area [78]. Prior to this study, it was assumed that puffins overwintered much farther away from the sea ice edge and did not utilize that habitat during the non-breeding season.

**Fig 5:**
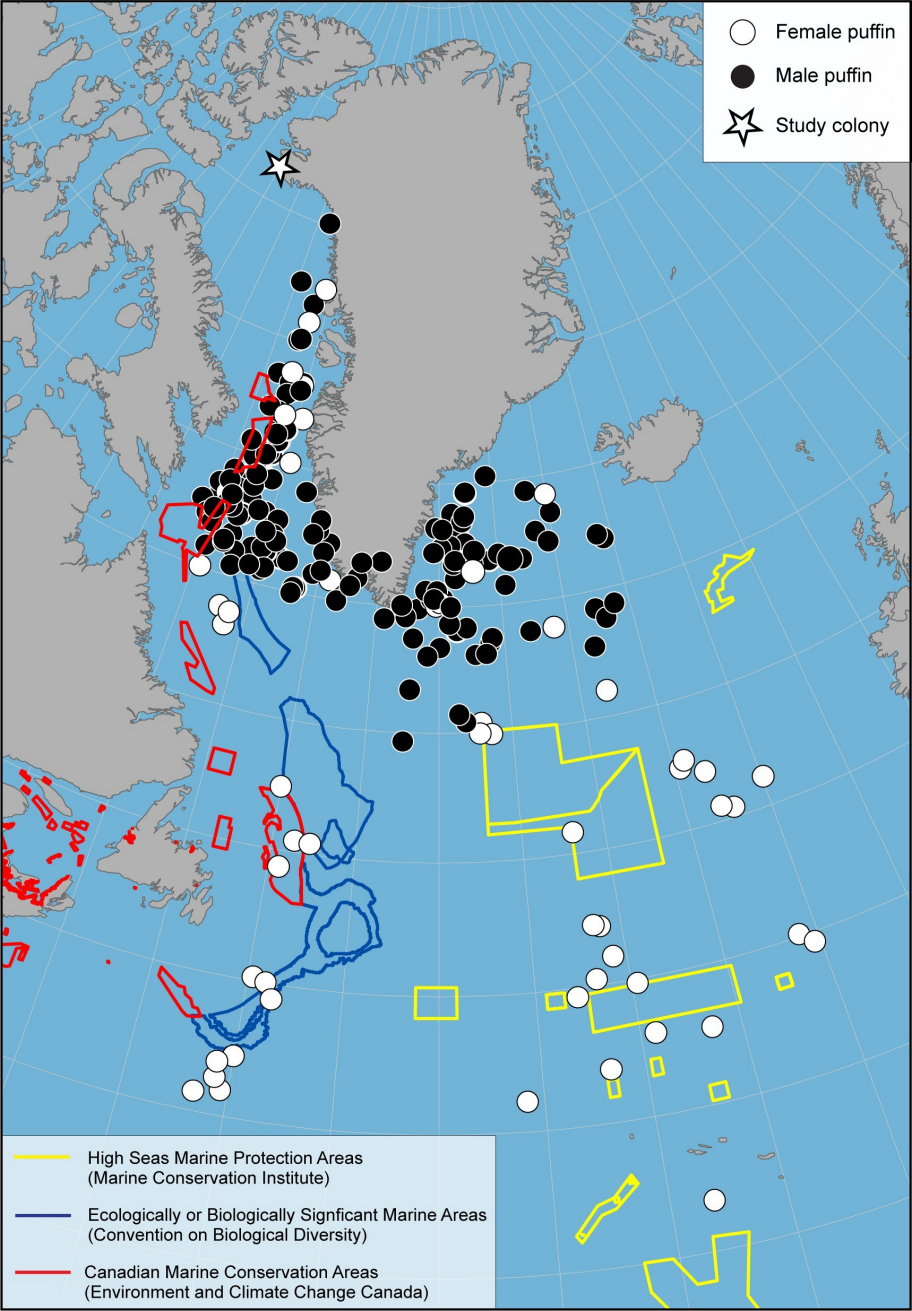
Puffin use of marine conservation areas. Tri-monthly median locations of Atlantic Puffins from northwest Greenland and marine conservation areas (of varying degrees of protection) in the North Atlantic [84–88]. Country boundaries from [28].

Puffins from northwest Greenland utilized limited marine conservation areas of the North Atlantic during migration, with phenology and use areas differing by sex. From October through December male puffins utilized portions of three Canadian conservation areas in the Davis Strait, but starting in February they utilized areas without conservation status off southeast Greenland (Fig 5). At-sea surveys are rare for southeast Greenland, but multi-colony studies have shown that the area is rarely used by seabirds during this period (e.g., black-legged kittiwake *Rissa tridactyla* [57], thick-billed murre *Uria lomvia* [42], little auk *Alle alle* [79], Atlantic puffin [47]). In contrast, female puffins traveled farther south and utilized Canadian conservation areas and ecologically and biologically significant marine areas (EBSA) of the western North Atlantic and marine protected areas (MPA) of the high seas along the mid-Atlantic, areas known to have large numbers of wintering seabirds [80–83].

### Body size and climate as potential migratory drivers

The substantial differences in migratory phenology, route, and wintering area between male and female puffins in this study may be driven by the observed difference in body size. For the male puffins that had complete migration routes in our study (*n* = 6), their body mass was on average 157 g greater (35.6% of mean female body mass) than the two female puffins with complete migrations (Table 1). It is worth mentioning, however, that the body mass of these two females (426 and 456 g) was low compared to other non-tagged female puffins from the same study population (mean = 526 ± 51.3, range 426–611, *n* = 22; Table 1 in [15])). For animals in general, and male puffins in particular, a larger body size may influence their ability to remain farther north during the non-breeding season in areas with colder temperatures and shorter days compared to individuals that breed farther south in their geographic distribution. For example, the fasting endurance hypothesis, one of several possible mechanisms proposed for within-species body size variation as described by Bergmann’s Rule, suggests that increased body size is favored in animals that live in more unpredictable and seasonal environments [89,90]. The larger body size may allow for greater fat storage and a lower weight-specific metabolic rate, which can increase survival during periods of limited food availability or stress [90].

The resting metabolic rate (RMR) and heat loss in seabirds increases as water and air temperature decrease, thus smaller individuals lose heat faster than larger individuals [91]. Within alcids, heat loss also rapidly increases as water temperature decreases [91]. Male puffins from northwest Greenland overwintered in regions with generally colder monthly mean SST compared to other puffin populations, and in areas as much as 10° C cooler than the two females from this study (Fig 4), likely resulting in a much higher RMR and daily energy expenditure (DEE). Similar results were noted by Fayet et al. [50], who also found that puffins wintering in colder waters (and higher latitudes) had higher DEE and spent more time foraging than birds wintering farther south in warmer waters. In our study, the increased energy used in flight by small female puffins to migrate nearly twice as far as males may thus be negated by the effect of warmer waters on RMR, in addition to less intra-specific competition for prey. Pelletier et al. [92] found similar results for northern gannets *Morus bassanus*, where the energetic costs of individuals that migrated much farther to warmer waters were outweighed by the benefits of lower costs in foraging and thermoregulation. Further research is warranted to explore how puffin body size and sex may interact with physiological constraints relative to overwintering location. Additionally, future use of geolocators with the ability to record temperature is suggested as temperature data assimilated with geolocation data has been shown to enhance the accuracy of the resulting positions [93,94].

## Conclusion

Puffins in northwest Greenland demonstrated unique migratory strategies when compared with all other puffin colonies studied to date. Whether these strategies are specific to northwest Greenland is unknown, and further research is needed to both increase the sample size in our study area, particularly among females, and to determine if similar patterns are observed among other high Arctic puffin colonies. The role larger body size may play in influencing overwintering location is similarly unknown, but is likely related to survival and environmental constraints, and should be further studied in puffins as well as other clinal seabird species with a similar latitudinal distribution. As Arctic sea ice continues to diminish and SST continues to warm, it is probable that puffins of the high Arctic may respond by shifting (or perhaps have already shifted) their non-breeding spatial distribution in the North Atlantic. To what degree the sexes may respond differently to these changes requires further study.
